# Supplementary material for: Altered microRNA Transcriptome in Cultured Human Liver Cells upon Infection with Ebola Virus
Source: Int J Mol Sci. 2021 Apr 6;22(7):3792. doi: 10.3390/ijms22073792 (PMC8038836; doi:10.3390/ijms22073792)
Supplement: Supplementary file 1 [file ijms-22-03792-s001.zip › Supplementary_File/D_ KEGG_Analysis_Results/16-30nt_pathway_Mayinga-24h-Huh7_vs_Control-24h-Huh7_down.mature_mirna_targets/hsa_pathwayResult.html]

| PathwayID | Definition | OriginalWebSite | Fisher-Pvalue | SelectionCounts | SelectionSize | Count | Size | FDR | Enrichment\_Score | GeneRatio | Genes |
| --- | --- | --- | --- | --- | --- | --- | --- | --- | --- | --- | --- |
| hsa04922 | Glucagon signaling pathway - Homo sapiens (human) | http://www.genome.jp/kegg-bin/show\_pathway?hsa04922+815+84699+2033+2308+5562 | 3.624115e-03 | 5 | 77 | 106 | 7906 | 4.774478e-01 | 2.440798 | 0.064935 | CAMK2A//CREB3L3//EP300//FOXO1//PRKAA1 |
| hsa04931 | Insulin resistance - Homo sapiens (human) | http://www.genome.jp/kegg-bin/show\_pathway?hsa04931+84699+2308+8473+5562+5588 | 3.926820e-03 | 5 | 77 | 108 | 7906 | 4.774478e-01 | 2.405959 | 0.064935 | CREB3L3//FOXO1//OGT//PRKAA1//PRKCQ |
| hsa04024 | cAMP signaling pathway - Homo sapiens (human) | http://www.genome.jp/kegg-bin/show\_pathway?hsa04024+23439+627+815+1128+84699+2033+2904 | 4.904299e-03 | 7 | 77 | 216 | 7906 | 4.774478e-01 | 2.309423 | 0.090909 | ATP1B4//BDNF//CAMK2A//CHRM1//CREB3L3//EP300//GRIN2B |
| hsa04918 | Thyroid hormone synthesis - Homo sapiens (human) | http://www.genome.jp/kegg-bin/show\_pathway?hsa04918+23439+84699+493869+7849 | 5.717938e-03 | 4 | 77 | 74 | 7906 | 4.774478e-01 | 2.242761 | 0.051948 | ATP1B4//CREB3L3//GPX8//PAX8 |
| hsa05034 | Alcoholism - Homo sapiens (human) | http://www.genome.jp/kegg-bin/show\_pathway?hsa05034+627+84699+2775+2904+8520+79885 | 8.852777e-03 | 6 | 77 | 184 | 7906 | 5.664020e-01 | 2.052920 | 0.077922 | BDNF//CREB3L3//GNAO1//GRIN2B//HAT1//HDAC11 |
| hsa05016 | Huntington disease - Homo sapiens (human) | http://www.genome.jp/kegg-bin/show\_pathway?hsa05016+627+84699+2033+2904+4697+5434 | 1.105165e-02 | 6 | 77 | 193 | 7906 | 5.664020e-01 | 1.956573 | 0.077922 | BDNF//CREB3L3//EP300//GRIN2B//NDUFA4//POLR2E |
| hsa05030 | Cocaine addiction - Homo sapiens (human) | http://www.genome.jp/kegg-bin/show\_pathway?hsa05030+627+84699+2904 | 1.187070e-02 | 3 | 77 | 49 | 7906 | 5.664020e-01 | 1.925524 | 0.038961 | BDNF//CREB3L3//GRIN2B |
| hsa04916 | Melanogenesis - Homo sapiens (human) | http://www.genome.jp/kegg-bin/show\_pathway?hsa04916+815+84699+2033+2775 | 1.667166e-02 | 4 | 77 | 101 | 7906 | 6.960416e-01 | 1.778021 | 0.051948 | CAMK2A//CREB3L3//EP300//GNAO1 |
| hsa04151 | PI3K-Akt signaling pathway - Homo sapiens (human) | http://www.genome.jp/kegg-bin/show\_pathway?hsa04151+627+1128+131873+84699+1944+3566+5562+7529 | 2.128544e-02 | 8 | 77 | 354 | 7906 | 7.111143e-01 | 1.671917 | 0.103896 | BDNF//CHRM1//COL6A6//CREB3L3//EFNA3//IL4R//PRKAA1//YWHAB |
| hsa00590 | Arachidonic acid metabolism - Homo sapiens (human) | http://www.genome.jp/kegg-bin/show\_pathway?hsa00590+8529+4051+493869 | 2.322988e-02 | 3 | 77 | 63 | 7906 | 7.111143e-01 | 1.633953 | 0.038961 | CYP4F2//CYP4F3//GPX8 |
| hsa04725 | Cholinergic synapse - Homo sapiens (human) | http://www.genome.jp/kegg-bin/show\_pathway?hsa04725+815+1128+84699+2775 | 2.341993e-02 | 4 | 77 | 112 | 7906 | 7.111143e-01 | 1.630414 | 0.051948 | CAMK2A//CHRM1//CREB3L3//GNAO1 |
| hsa04720 | Long-term potentiation - Homo sapiens (human) | http://www.genome.jp/kegg-bin/show\_pathway?hsa04720+815+2033+2904 | 2.726318e-02 | 3 | 77 | 67 | 7906 | 7.277685e-01 | 1.564424 | 0.038961 | CAMK2A//EP300//GRIN2B |
| hsa05031 | Amphetamine addiction - Homo sapiens (human) | http://www.genome.jp/kegg-bin/show\_pathway?hsa05031+815+84699+2904 | 2.832632e-02 | 3 | 77 | 68 | 7906 | 7.277685e-01 | 1.547810 | 0.038961 | CAMK2A//CREB3L3//GRIN2B |
| hsa04360 | Axon guidance - Homo sapiens (human) | http://www.genome.jp/kegg-bin/show\_pathway?hsa04360+815+1944+10509+57715+90249 | 3.126638e-02 | 5 | 77 | 181 | 7906 | 7.459265e-01 | 1.504922 | 0.064935 | CAMK2A//EFNA3//SEMA4B//SEMA4G//UNC5A |
| hsa03020 | RNA polymerase - Homo sapiens (human) | http://www.genome.jp/kegg-bin/show\_pathway?hsa03020+5434+171568 | 3.628893e-02 | 2 | 77 | 31 | 7906 | 7.569779e-01 | 1.440226 | 0.025974 | POLR2E//POLR3H |
| hsa04068 | FoxO signaling pathway - Homo sapiens (human) | http://www.genome.jp/kegg-bin/show\_pathway?hsa04068+1032+2033+2308+5562 | 3.852881e-02 | 4 | 77 | 131 | 7906 | 7.569779e-01 | 1.414214 | 0.051948 | CDKN2D//EP300//FOXO1//PRKAA1 |
| hsa04728 | Dopaminergic synapse - Homo sapiens (human) | http://www.genome.jp/kegg-bin/show\_pathway?hsa04728+815+84699+2775+2904 | 3.852881e-02 | 4 | 77 | 131 | 7906 | 7.569779e-01 | 1.414214 | 0.051948 | CAMK2A//CREB3L3//GNAO1//GRIN2B |
| hsa03018 | RNA degradation - Homo sapiens (human) | http://www.genome.jp/kegg-bin/show\_pathway?hsa03018+7832+131870+10140 | 4.145370e-02 | 3 | 77 | 79 | 7906 | 7.691964e-01 | 1.382437 | 0.038961 | BTG2//NUDT16//TOB1 |
| hsa05203 | Viral carcinogenesis - Homo sapiens (human) | http://www.genome.jp/kegg-bin/show\_pathway?hsa05203+84699+2033+79885+6722+7529 | 4.579223e-02 | 5 | 77 | 201 | 7906 | 8.049792e-01 | 1.339208 | 0.064935 | CREB3L3//EP300//HDAC11//SRF//YWHAB |
